# Metabolic Changes Reveal the Development of Schistosomiasis in Mice

**DOI:** 10.1371/journal.pntd.0000807

**Published:** 2010-08-31

**Authors:** Junfang Wu, Wenxin Xu, Zhenping Ming, Huifen Dong, Huiru Tang, Yulan Wang

**Affiliations:** 1 State Key Laboratory of Magnetic Resonance and Atomic and Molecular Physics, Wuhan Center for Magnetic Resonance, Wuhan Institute of Physics and Mathematics, Chinese Academy of Sciences, Wuhan, People's Republic of China; 2 Graduate School of Chinese Academy of Sciences, Beijing, People's Republic of China; 3 Department of Medical Parasitology, School of Basic Medical Science, Wuhan University, Wuhan, People's Republic of China; George Washington University Medical Center, United States of America

## Abstract

Schistosomiasis is a parasitic zoonosis caused by small trematode worms called schistosomes, amongst which *Schistosoma japonicum* (*S. japonicum*) is endemic in Asia. In order to understand the schistosome-induced changes in the host metabolism so as to facilitate early diagnosis of schistosomiasis, we systematically investigated the dynamic metabolic responses of mice biofluids and liver tissues to *S. japonicum* infection for five weeks using ^1^H NMR spectroscopy in conjunction with multivariate data analysis. We were able to detect schistosomiasis at the third week post-infection, which was one week earlier than “gold standard” methods. We found that *S. japonicum* infection caused significant elevation of urinary 3-ureidopropionate, a uracil catabolic product, and disturbance of lipid metabolism, stimulation of glycolysis, depression of tricarboxylic acid cycle and disruption of gut microbiota regulations. We further found that the changes of 3-ureidopropionate and overall metabolic changes in both urinary and plasma samples were closely correlated with the time-course of disease progression. Furthermore, such changes together with liver tissue metabonome were clearly associated with the worm-burdens. These findings provided more insightful understandings of host biological responses to the infection and demonstrated that metabonomic analysis is potentially useful for early detection of schistosomiasis and comprehension of the mechanistic aspects of disease progression.

## Author Summary

Schistosomiasis is an infectious disease resulting from the infection of parasitic trematode worms called schistosomes. About 600 million people are currently exposed to schistosomiasis and 200 million people are infected in about 76 countries. Current diagnostic methods are unable to detect schistosomiasis at its early stages and thus are incapable of preventing disease causing further complications. In order to understand the effects of schistosome infection on hosts' biochemistry associated with disease progression in a holistic fashion and detect the infection at the early stage, we systematically investigated the metabolite composition (metabonome) changes in mice biofluids and liver tissues induced by *Schistosoma japonicum* using NMR spectroscopy. We detected infection-induced mice metabonomic alterations at three weeks post-infection, a week earlier than traditional methods. We found that the infection-caused elevation of urinary 3-ureidopropionate was not only associated with disease progression but also worm burden. We further found that overall metabonomic changes were also closely associated with disease progression, and our methods were capable of distinguishing different levels of worm burden at week five post-infection. Our findings provided further understandings in host responses to the infection and demonstrated metabonomics as a potentially useful tool for early diagnosis of *S. japonicum* infections.

## Introduction

Schistosomiasis is a chronic parasitic disease caused by infection with schistosomes. As one of the most infectious species, *Schistosoma japonicum* is mainly endemic in Asia with over 1 million infected individuals and about 46 million people at risk in China, the Philippines and Indonesia [1]–[3]. During schistosomiasis progression, schistosomes mature to adults in the hepatic circulation and then in pairs migrate to inhabit in the mesenteric veins, where they mate and lay a large number of eggs in the vessels of the intestinal wall. Consequently, schistosomiasis causes diarrhea, fatigue, anemia at the early stage of infection, and portal vein hypertension syndrome, ascites and liver fibrosis at the later stages [1]. Currently, schistosomiasis is diagnosed using the Kato-Katz technique by detecting eggs in feces under microscope [4], or with immunological approaches by detecting soluble antigens secreted from the hatching-eggs *via* the antigen-antibody reaction [5]–[6]. However, such methods are not suitable for early diagnosis and the adverse effects associated with the deposition of schistosome eggs would have already occurred when diagnosis were made. Therefore, development of early diagnostic methods is in urgent need so as to treat patients timely to prevent clinical complications. Understanding the dynamic responses of the hosts with schistosomiasis in the systems level is important to provide insights into the mechanisms underlying disease progression and thus could be potentially useful for early diagnosis of schistosomiasis.

Previous work has examined the schistosomiasis-caused alterations in the transcription and protein levels. Reductions in biologically active albumin mRNA and increased type I procollagen mRNA were observed in the liver of *S. mansoni* infected mice 6 weeks post-infection [7]. However, no such changes were noted at the earlier stages of infection [7]. The expressions of proteins associated with structural components (procollagen VI, keratin and actin), the stress responses (heat shock proteins, chaperones) were significantly promoted [8], which may be relevant to the infection-caused liver fibrosis and schistosomes' uptake of host proteins onto their tegument during development [9]. Furthermore, *S. mansoni* infection caused significant increases in the activities of pyruvate kinase and phosphofructokinase but marked reductions in the activities of citrate synthase, glycogen phosphorylase, glucose-6-phosphate dehydrogenase, carbamoyl phosphate synthetase and ornithine carbamoyltrasferase [10]–[11]. Such observations were consistent with recent proteomic results that *S. mansoni* infection (for 8 weeks) caused remarkable decreases in the expression of host liver enzymes associated with the Krebs cycle, fatty acid cycle, urea cycle, amino acid metabolism and catabolism, amongst which the expression of malic enzyme was decreased 15-folds by infection [8]. However, it is not clear thus far whether such systems responses are generic for infections by other schistosome species and what are the host systems responses at the early stage of infections.

The analysis of host metabolite composition (i.e., metabonome) is a well suited approach to understand the holistic metabolic responses to infections since metabonomics is a branch of science concerned with the metabolite composition of biological systems and its dynamic responses to both endogenous and exogenous stimuli [12]–[14]. As a powerful holistic analytical approach, metabonomics has already been widely applied in studies of disease pathogenesis [15]–[17], drug toxicity [18]–[19] and in the environmental [20]–[21] and nutritional sciences [22]–[23]. This approach has also been successfully applied in parasitological studies with comprehensive characterizations of the host metabolic responses to infections by several parasites, such as *Trypanosoma brucei brucei* [24], *Plasmodium berghei* [25] and *Echinostoma caproni* [26]–[27]. The results also showed that schistosomal infections led to suppression of the hosts' Krebs cycles, disruption of amino acid metabolism, liver injuries and disturbances of the gut microbiota [28]–[29]. A recent study revealed that *S. mansoni* infection resulted in significant metabolic alterations in a range of mouse tissues [30]. Metabolic alterations were also comprehensively studied for hamster models co-infected with *S. japonicum* and *Necator americanus* [31]. However, the previous investigations were all based on a well-established late-stage schistosomal infection model. The dynamic metabolic responses associated with progression of infection remained to be elucidated.

In this work, we systematically investigated the time-course metabonomic changes in urine and blood plasma of the *S. japonicum* infected mice over 5 weeks and liver tissues at the fifth week post-infection using ^1^H NMR spectroscopy and multivariate data analysis. The main objectives are to define the host metabonomic responses to infection at the early stages and their dynamic changes during the disease progression, which are of potential importance for early diagnosis and prognostic understandings of schistosomiasis.

## Materials and Methods

### Parasite, host and infection

A total of 60 female pathogen free BALB/c mice, about 8 weeks old weighing 20±2 g, were purchased from the animal laboratory center of Wuhan university (China), and housed in groups of 5 in plastic cages under environmentally-controlled conditions (temperature: 18∼22°C; humidity: 40∼70%; light-dark cycle: 12–12 h). Mice had free access to rodent food and water. After 3 weeks of acclimatization, half of the mice (n = 30) were infected with 80±2 *S. japonicum* cercariae, each *via* shaved abdominal skin. The cercariae were obtained from infected *O. hupensis* (Anhui) after exposure to artificial light. The rest of the mice served as controls.

### Ethics statement

All experimental procedures were performed according to the National Guidelines for Experimental Animal Welfare (MOST of People's Republic of China, 2006) and were approved by the Animal Welfare Committee of Wuhan University (Permission No. SYXK (E) 2008–0013).

### Sample collection, *S. japonicum* worm burden and histology

Plasma and urine samples were collected one day before infection and after infection for five weeks on a weekly basis. Sample collection was carried out between 08:30–11:30 in order to avoid potential metabolic variations due to diurnal cycle. Blood samples (70∼80 µl) were collected from the orbital venous plexus and transferred to Eppendorf tubes containing 5 µl sodium heparin, followed by centrifugation at 3000 g for 10 mins. The supernatant (∼30 µl) was transferred into 0.5 ml Eppendorf tubes, immediately immersed in liquid nitrogen, and stored at −80°C. Urine samples (50∼400 µl) were collected in empty plastic boxes by gently massaging the abdomen of mice and transferred into Eppendorf tubes, stored at −80°C.

Half of the mice (15 control and 15 infected mice) were sacrificed at 5 weeks post-infection by cervical dislocation and the remaining mice were kept for a separate experiment. Plasma at sacrifice was divided into two portions with one portion kept for NMR analysis and the other for clinical biochemistry analysis. The adult schistosomes were isolated by perfusion *via* the heart with saline solution containing heparin. The worms in the portal vein and the mesenteric veins were pushed out gently with a dissecting needle. All harvested *S. japonicum* worms were sexed and counted for worm burden assessments. The middle lobe of liver was excised and immediately snap-frozen in liquid nitrogen and stored at −80°C for ^1^H High Resolution Magic Angel Spinning (HR MAS) NMR analysis. Another small portion of the liver was stored in 10% formalin solution for histological assessments where tissue samples were sectioned into 5 µm slices and stained with H&E and examined under a light microscope (BRX-51, Leica, Germany).

### ^1^H NMR spectroscopy

Urine samples were prepared by adding D_2_O into urine (50∼400 µl) to make a final volume of 500 µl. Then the liquid was mixed with 50 µl Na^+^/K^+^ buffer (K_2_HPO_4_/NaH_2_PO_4_ in D_2_O, 1.5M, pD 7.4) [32], containing 0.01% sodium 3-trimethylsilyl (2,2,3,3-^2^H4) propionate (TSP) for chemical shift reference. After being vortexed and centrifuged at 10000 g, 4°C, supernatant of 500 µl was transferred into 5 mm NMR tubes. The urinary ^1^H NMR spectra were acquired at 298 K from a Bruker AVIII 600 MHz NMR spectrometer (Bruker Biospin, Germany) equipped with a cryogenic probe, operating at 600.13 MHz proton frequency. The plasma samples were prepared by mixing 30 µl plasma with 30 µl saline solution containing 95% D_2_O, and 30 mM phosphate buffer (pD 7.4). The mixed liquid was transferred into 1.7 mm micro NMR tubes. ^1^H NMR spectra of plasma were recorded at 298 K on a Bruker AV∏ 500 NMR spectrometer, operating at 500.13 MHz proton frequency with a broad band inverse detection probe. Liver samples (about 15∼20 mg) were rinsed with 0.9% saline (D_2_O) and placed in a 4 mm zirconia rotor with a spin rate of 2200 Hz. HR MAS ^1^H NMR spectra of liver tissues were acquired at 283 K on a Varian INOVA-600 spectrometer equipped with a Varian nanoprobe, operating at 599.81 MHz proton frequency.

A standard water suppressed one dimensional NMR experiment using sequence [recycle delay −90°−*t_1_*−90°−*t_m_*−90°-acquisition] was employed for urine [28]. A spin relaxation edited-water saturated ^1^H NMR experiment using Carr-Purcell-Meiboom-Gill (CPMG) pulse sequence was performed for both plasma and liver tissues. A total spin-spin relaxation delay of 70 ms and 400 ms was used for plasma and liver tissues respectively. The 90° pulse length was adjusted to 10 µs. A total of 256 scans were accumulated into 32 k data points, with a spectral width of 20 ppm for plasma and liver samples, and 32 scans were recorded for urine samples. For spectral assignment purposes, two dimensional (2D) NMR spectra (^1^H-^1^H COSY and TOCSY, ^1^H-^13^C HSQC and HMBC) were acquired on selected samples utilizing standard acquisition parameters [18], [33]–[34].

### Data reduction and multivariate pattern recognition analysis

^1^H NMR spectra were corrected for phase and baseline distortion, and referenced manually using TOPSPIN package (V2.0, Bruker Biospin, Germany). Spectra were segmented into integral regions of 0.002 ppm for urine and 0.004 ppm for plasma and liver using the AMIX package (V3.8, Bruker Biospin, Germany). The distorted water regions were removed to eliminate the effects of water suppression prior to normalization of the data to the total sum intensity of the spectrum.

SIMCA-P^+^ software package (V.12, Umetrics, Sweden) was employed for multivariate data analysis. Principal component analysis (PCA) was performed by using a mean-centered NMR data to identify general trends and outliers. A supervised multivariate data analysis tool, orthogonal-projection to latent structure discriminant analysis (O-PLS-DA) [35]–[36], was employed with the Pareto scaling method [37]. All models were cross validated using a 7-fold method [38]. In supervised pattern recognition method, validation of model is crucial for interpretation of data and hence all models here have been rigorously evaluated with permutation tests (permutation numbers = 200) [39]–[40]. In order to facilitate interpretation of the results, back-transformation of the loadings was performed as described previously [41] and plotted with color-coded coefficients for each variable using in-house developed MATLAB scripts.

### Clinical biochemistry analysis

The clinical biochemistry of serum was measured using an automatic biochemistry analyzer. Independent *t-*tests were conducted using SPSS 12.0 software and expressed as mean ± SD.

## Results

### Parasitemia, histology and clinical chemistry

On average, 42 live *S. japonicum* worms were found (with standard deviation of 12) in the infected mice but with no significant bodyweight differences between the control and infected animals. Histopathological examinations of liver from the infected mice (5 weeks post-infection) showed grayish irregular nodules and marked schistosomal hepatic lesions (Fig. S1). Clinical serum chemistry results (Table 1) indicated that *S. japonicum* infection led to significant increases in the activities of alanine aminotransferase (ALT) (*ca.* 5 folds), aspartate aminotransferase (AST) (>1 fold), and their ratio (ALT/AST). Infection also caused significant increases in the levels of globulin and decreases in the levels of albumin, alkaline phosphatase, triglyceride and the albumin-to-globulin ratio.

**Table 1:** Clinical chemistry data for the control and *S. japonicum* infected mice at week 5 post-infection†.

	5 weeks post-infection		
	control	Infection	Significance
ALT (IU/L)	61.0±20.4	358.7±199.6***	↑
AST (IU/L)	166.3±54.7	381.4±152.1***	↑
ALT/AST	0.4±0.10	0.9±0.3***	↑
ALB (IU/L)	41.6±1.42	38.0±4.0**	↓
Globulin (IU/L)	17.1±3.0	22.1±3.3***	↑
A/G	2.5±0.3	1.8±0.4***	↓
TP (IU/L)	58.7±3.7	60.1±3.0	-
ALP (IU/L)	108.9±16.2	72.7±30.0***	↓
Glucose (mmol/L)	6.4±0.8	5.7±1.1	-
Chol (mmol/L)	2.6±0.4	2.4±0.2	-
TG (mmol/L)	1.2±0.4	0.8±0.3***	↓

**†:** Values are expressed as means ± S.D. Statistics: (**)*p*<0.01, (***)*p*<0.001 (n = 15 for each group, except for the TG and ALP of the non-infected group where n = 14 due to insufficient sample volume). Keys: ALT (alanine aminotransferase), AST (aspartate aminotransferase), ALP (alkaline phosphatase), TP (total protein), ALB (Albumin), Chol (cholesterol), TG (Triglyceride).

### ^1^H NMR spectra of plasma, urine and liver tissues

**Figure 1:**
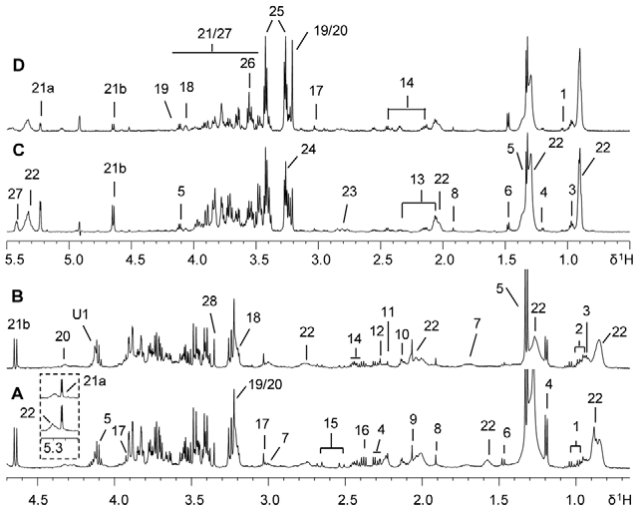
^1^H NMR spectra of plasma and liver tissue from control and *S. japonicum* infected mice. Typical 500 MHz ^1^H (CPMG) NMR spectra of plasma obtained from a non-infected BALB/c mouse (A) and a mouse infected with *S. japonicum* for 5 weeks (B). Typical 600 MHz ^1^H HRMAS (CPMG) NMR spectra of intact liver tissue obtained from a non-infected mouse (C) and a mouse infected with *S japonicum* for 5 weeks (D). Keys: 1, valine; 2, leucine; 3, isoleucine; 4, D-3-hydroxybutyrate; 5, lactate; 6, alanine; 7, lysine; 8, acetate; 9, *N*-acetyl-glycoprotein; 10, methionine; 11, acetone; 12, acetoacetate; 13, glutamate; 14, glutamine; 15, citrate; 16, pyruvate; 17, creatine; 18, choline; 19, phosphorylcholine; 20, glyceryl phosphorylcholine; 21a, α-glucose; 21b, β-glucose; 22, lipids; 23, aspartate; 24, trimethylamine-*N*-oxide; 25, taurine; 26, glycine; 27, glycogen; 28; *scyllo*-inositol; U1, unknown1.

**Figure 2:**
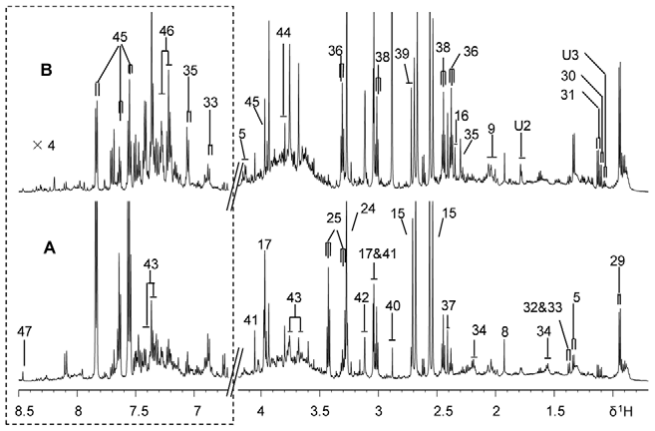
^1^H NMR spectra of urine samples from control and *S. japonicum* infected mice. Typical 600 MHz ^1^H NMR spectra of urine samples obtained from a non-infected BALB/c mouse (A) and a mouse infected with *S. japonicum* for 5 week (B). The spectral region, δ 6.7–8.5, was vertically expanded 4 times compared with the aliphatic region (δ 0.7–4.2). Keys: 5, lactate; 8, acetate; 9, *N*-acetyl-glycoproteins; 15, citrate; 16, pyruvate; 17, creatine; 24, trimethylamine-*N*-oxide; 25, taurine; 29, 2-keto-isocaproate; 30, 2-keto-3-methyl-valerate; 31, 2-keto-isovalerate; 32, 2-hydroxyisobutyrate; 33, 2-(4-hydroxyphenyl) propanoic acid; 34, adipate; 35, 4-cresol glucuronide; 36, 3-ureidopropionate; 37, succinate; 38, 2-oxo-glutarate; 39. dimethylamine; 40, trimethylamine; 41, creatinine; 42, malonate; 43, phenylacetylglycine; 44, guanidinoacetate; 45, hippurate; 46, indoxylsulfate; 47, formate; U2,unknown2; U3, unknown3.

^1^H NMR spectra of mice plasma, liver tissues (Fig. 1) and urine (Fig. 2) samples contained rich metabolite information with all NMR resonances assigned according to literature data [34], [42]–[43] and further confirmed with a catalogue of 2D NMR spectra. Glucose, lipoproteins, citrate, creatine and a range of amino acids were detected in the plasma and intact liver tissues from both control and infected BALB/c mice (Fig. 1). Visual inspection of the plasma spectra revealed that the infected samples (Fig. 1B) contained higher levels of *N*-acetyl-glycoproteins together with lower levels of lipids, citrate and alanine than controls (Fig. 1A). The metabolic profiles of liver tissues of the infected mice (Fig. 1D) had lower levels of glucose and glycogen accompanied with higher levels of choline metabolites, such as phosphorylcholine (PC) and glyceryl phosphorylcholine (GPC), and alanine than controls (Fig. 1C).

^1^H NMR spectra of urine from infected mice (Fig. 2B) showed obvious elevated levels of 2-keto-isocaproate, 2-keto-3-methyl-valerate, 2-keto-isovalerate, pyruvate, 4-cresol glucuronide, 3-ureidopropionate (3-UP), dimethylamine (DMA), trimethylamine (TMA), phenylacetylglycine (PAG) and alleviated levels of adipate, taurine and hippurate compared with the controls (Fig. 2A). To further obtain the detailed metabonomic differences, we employed multivariate data analysis approaches.

### Metabolic variations associated with *S. japonicum* infection

Initial PCA of the mean-centered NMR spectral data from plasma of the *S. japonicum* infected mice and corresponding controls showed that one control sample containing markedly low levels of lipoproteins appeared to cluster closely with the infected group (data not shown). One sample from the infected group was associated with the control group at all time points probably because it had significantly low worm burden with worm count of only 18. We therefore removed these two animals from subsequent data analysis in order to avoid possible confusions.

**Figure 3:**
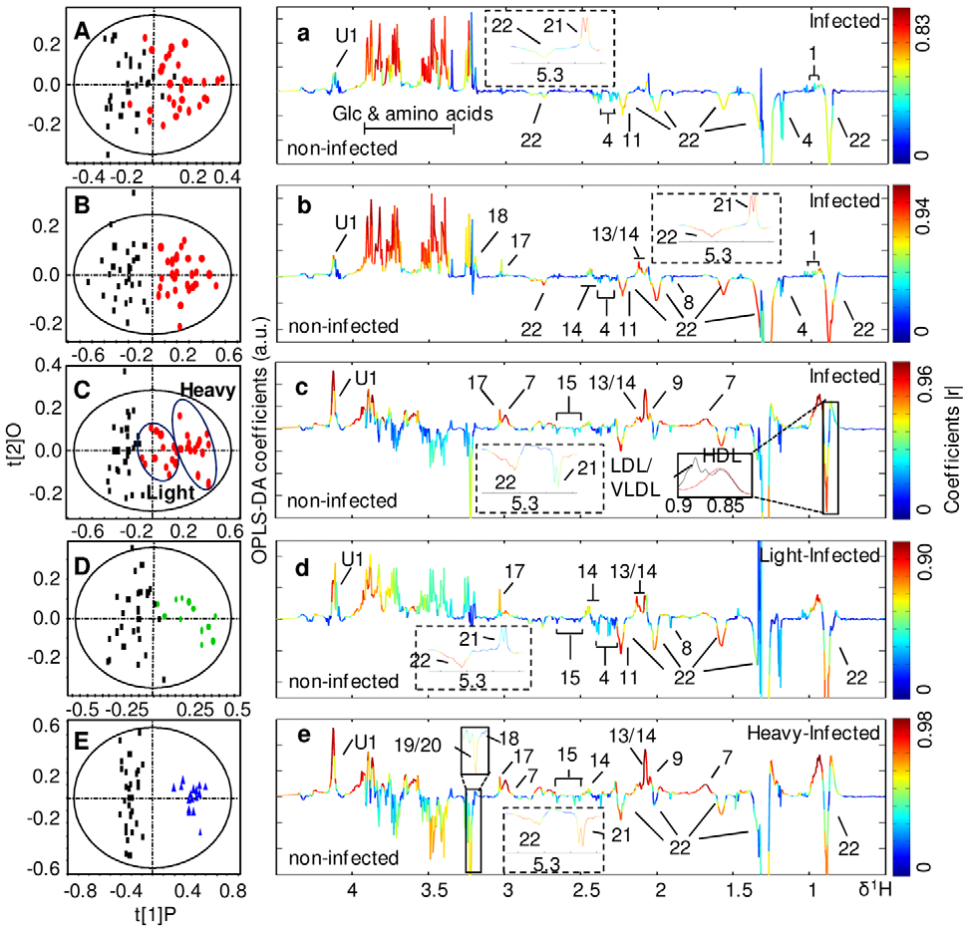
O-PLS-DA comparison between NMR spectra of plasma from the *S. japonicum* infected mice and corresponding controls. Cross-validated scores (left) and coefficient-coded loadings (right) plots for the comparative O-PLS-DA of plasma data for non-infected and *S. japonicum*-infected mice at different time points. Non-infected (black squares) vs infected (red dots) mice 3 weeks post-infection (A), 4 weeks post-infection (B) and 5 weeks post-infection (C). Non-infected vs lightly infected mice (green) (D) and heavily infected mice (blue) (E) at week 5 post-infection. The colored scale is for coefficients being indicative to the significance of metabolite contributions to the differentiation between classes. Keys: 1, valine; 2, leucine; 3, isoleucine; 4, D-3-hydroxybutyrate; 5, lactate; 6, alanine; 7, lysine; 8, acetate; 9, *N*-acetyl-glycoprotein; 10, methionine; 11, acetone; 12, acetoacetate; 13, glutamate; 14, glutamine; 15, citrate; 16, pyruvate; 17, creatine; 18, choline; 19, phosphorylcholine; 20, glyceryl phosphorylcholine; 21a, α-glucose; 21b, β-glucose; 22, lipid; 23, aspartate; 24, trimethylamine-*N*-oxide; 25, taurine; 26, glycine; 27, glycogen; 28; *scyllo*-inositol; U1, unknown1.

**Figure 4:**
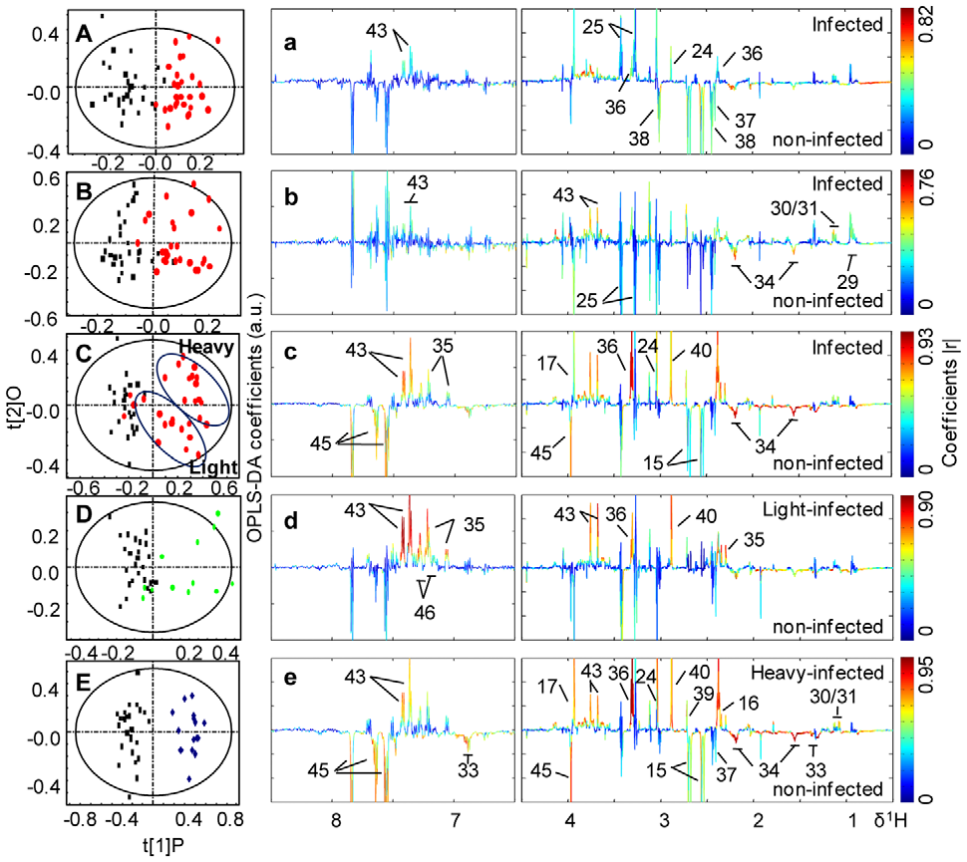
O-PLS-DA comparison between NMR spectra of urine from *S. japonicum* infected mice and corresponding controls. Cross-validated scores (left) and coefficient-coded loadings (right) plots for the comparative O-PLS-DA of urine data for non-infected and *S. japonicum*-infected mice at different time points. Non-infected (black boxes) vs infected (red dots) mice 3 weeks post-infection (A), at 4 weeks post-infection (B) and 5 weeks post-infection (C). Non-infected vs lightly infected mice (green) (D) and heavily infected mice (blue) (E) at week 5 post-infection. Keys: 29, 2-keto-isocaproate; 30, 2-keto-3-methyl-valerate; 31, 2-keto-isovalerate; 32, 2-hydroxyisobutyrate; 33, 2-(4-hydroxyphenyl) propanoic acid; 34, adipate; 35, 4-cresol glucuronide; 36, 3-ureidopropionate; 37, succinate; 38, 2-oxo-glutarate; 39. dimethylamine; 40, trimethylamine; 41, creatinine; 42, malonate; 43, phenylacetylglycine; 44, guanidinoacetate; 45, hippurate; 46, indoxylsulfate; 47, formate; U2; unknown2; U3, unknown3.

**Figure 5:**
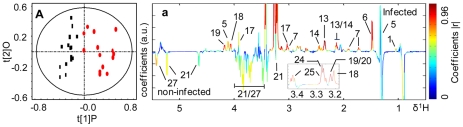
O-PLS-DA comparison between NMR spectra of liver tissue from the *S. japonicum* infected mice and controls. Cross-validated scores (left) and coefficient-coded loadings (right) plots for the comparative O-PLS-DA of the CPMG filtered ^1^H HRMAS NMR data of liver obtained from control (black boxes) and infected (red dots) mice at week 5 post-infection.

PCA trajectory (Fig. S2) demonstrated that the metabolic profiles of both mice plasma and urine samples had an association with the time course of *S. japonicum* infection and disease progression. The metabolic profiles obtained from the infected mice deviated from the corresponding controls from the third week post-infection onwards and such separations became more obvious as disease progression. In order to identify the metabolites associated with such separations, we further compared the metabolic profiles obtained from the infected mice and corresponding controls for all matched time points, including the pre-infection day, week 1, 2, 3, 4 and 5 post-infection, using O-PLS-DA strategy. The same strategy was utilized to analyze spectral data of liver tissues obtained from mice at week 5 post-infection. These O-PLS-DA models were validated using a 7-fold cross-validation strategy and rigorous permutation tests [39]–[40]. Judged from the values of R^2^X (goodness of fit) and Q^2^ (robustness of the models) (Tables 2 and 3) and permutation tests (Fig. S3; Fig S4), valid O-PLS-DA models were obtained for plasma and urine samples collected at week 3, 4 and 5 post-infection (Fig. 3A–C and Fig. 4A–C), and liver tissues at week 5 post-infection (Fig. 5). As noted, plasma (Fig. 3C) and urine (Fig. 4C) samples collected at week 5 post-infection were further separated into two subgroups. These subgroups were associated with the numbers of worm burden, including a group with a light-infection (average worms: 36.3±11.1) and the other a heavy-infection (average worms: 51±8.4, *p* = 0.02). Additional O-PLS-DA comparisons between the control group and the lightly-infected, the control and the heavily-infected groups were performed to assess the variation of metabolites at different infection levels in plasma and urine (Fig. 3 D, E and Fig. 4 D, E).

**Table 2:** Metabolites with significant contributions to the discrimination between non-infected and *S. japonicum*-infected mice derived from plasma and liver.

Metabolites (key)	Chemical shift (ppm)	Plasma R^2^X = 0.53 Q^2^ = 0.24	Plasma R^2^X = 0.53 Q^2^ = 0.64	Plasma R^2^X = 0.48 Q^2^ = 0.66	Plasma R^2^X = 0.52 Q^2^ = 0.60	Plasma R^2^X = 0.71 Q^2^ = 0.91	Liver R^2^X = 0.63 Q^2^ = 0.73
		inf vs con	inf vs con	inf vs con	light vs con	heavy vs con	inf vs con
		3weeks p.i.	4weeks p.i.	5weeks p.i.	5weeks p.i.	5weeks p.i.	5weeks p.i.
Acetate	1.91		−0.54†				
Acetone	2.23	−0.55	−0.74	−0.40	−0.64		
Alanine	1.48			−0.47		−0.60	+0.87
Aspartate	2.68, 2.82						+0.93
Choline	3.19		+0.60				+0.81
Citrate	2.52, 2.67			−0.59		−0.66	
Creatine	3.92		+0.69	+0.75	+0.71	+0.73	+0.74
D-3-Hydroxybutyrate	1.18	−0.42	−0.53				
Dihydrothymine	1.07		+0.37	+0.67		+0.75	
Glycine	3.55	+0.55	+0.75				
Glycogen	5.41						−0.70
Glucose	3.24	+0.83	+0.92	−0.47	+0.53	−0.78	−0.61
Glutamate	2.09		+0.66	+0.87	+0.87	+0.85	+0.95
Glutamine	2.45		+0.86	+0.73	+0.72	+0.81	+0.89
GPC + PC	3.21			−0.60		−0.72	+0.95
Lactate	1.33						+0.68
Lysine	1.70			+0.92	+0.75	+0.96	+0.87
Lipid fraction	0.89, 1.25, 1,57, 2.02, 2.23, 2.72, 5.30	−0.54	−0.80	−0.83	−0.80	−0.77	
*N*-acetyl-glycoprotein	2.07			+0.92		+0.92	
Pyruvate	2.37			−0.37		−0.52	
*Scyllo*-inositol	3.35		+0.38				
Taurine	3.42						+0.92
TMAO	3.27						+0.92
Valine	1.04	+0.48	+0.58	+0.38			+0.76
Unknown1	4.12	+0.59	+0.74	+0.96	+0.90	+0.98	

†Numbers are correlation coefficients obtained from O-PLS-DA.

+ indicates an increase in the concentration of metabolites in the infected group.

− indicates a decrease in the concentration of metabolites in the infected group.

p.i. stands for post-infection.

**Table 3:** Urinary metabolites with significant contributions to the discrimination between non-infected and *S. japonicum*-infected mice.

Metabolites (key)	Chemical shift (ppm)	R^2^X = 0.31	R^2^X = 0.36	R^2^X = 0.42	R^2^X = 0.30	R^2^X = 0.47
		Q^2^ = 0.53	Q^2^ = 0.46	Q^2^ = 0.56	Q^2^ = 0.60	Q^2^ = 0.89
		inf vs con	inf vs con	inf vs con	light vs con	heavy vs con
		3weeks p.i.	4weeks p.i.	5weeks p.i.	5weeks p.i.	5weeks p.i.
2-(4-hydroxyphenyl) propanoic acid	1.37, 3.57, 6.89, 7.20			−0.83		−0.9
2-Oxoglutarate	2.45, 3.01	−0.63†	−0.39			
2-hydroxyisobutyrate	1.36		−0.6	−0.77		−0.66
2-keto-3-methyl-valerate	1.10	+0.47	+0.49	+0.45		+0.52
2-keto-isocaproate	0.94, 2.1, 2.61		+0.42			
2-keto-isovalerate	1.13	+0.46	+0.62	+0.67		+0.72
3-Ureidopropionate	2.38	+0.46	+0.55	+0.86	+0.83	+0.93
4-cresol glucuronide	2.29, 7.06, 7.23		+0.60	+0.72	+0.8	+0.64
Acetate	1.92			−0.48		
Adipate	1.57, 2.20	−0.81	−0.68	−0.91	−0.75	−0.94
Citrate	2.56, 2.72	−0.68		−0.64		−0.8
Creatine	3.03,3.93	+0.4	−0.45	+0.66		+0.84
Dimethylamine	2.71		+0.61	+0.79	+0.58	+0.82
Fumarate	6.53		−0.51	−0.72		−0.62
Glycine	3.55			+0.5	+0.56	+0.52
Hippurate	7.55, 7.84			−0.73		−0.82
Indoxylsulfate	7.21, 7.27			+0.64	+0.8	+0.6
Lactate	1.33		+0.51			
Malonate	3.12			+0.49		+0.55
*N*-acetyl-glycoprotein	2.02	+0.7	+0.52	+0.76		+0.65
PAG	3.68 3.75,7.37, 7.43	+0.67	+0.54	+0.76	+0.9	+0.77
Pyruvate	2.34		+0.7	+0.77	+0.81	+0.72
Succinate	2.41	−0.41	−0.52	−0.39		
Taurine	3.27, 3.43	+0.48	−0.46	−0.51		
Trimethylamine	2.88	+0.5	+0.38	+0.73	+0.85	+0.83
TMAO	3.27	+0.53		+0.48		+0.59
Unknown 2	1.78		+0.55	+0.69		+0.78
Unknown 3	1.08			+0.65		+0.76

†Numbers are correlation coefficients obtained from O-PLS-DA.

+ indicates an increase in the concentration of metabolites in the infected group.

− indicates a decrease in the concentration of metabolites in the infected group.

p.i. stands for post-infection.

Color-coded coefficient plots (Fig. 3a–e and Fig. 4a–e) from O-PLS-DA revealed detailed mice metabolic changes induced by *S. japonicum* infection with coefficients summarized in Tables 2 and 3. The discrimination significance at the level of *p*<0.05 was determined for specific metabolites according to the test for the significance based on the Pearson product-moment correlation coefficients, where the absolute coefficient cutoff values (|r|) were 0.361 for models following 3, 4 and 5 weeks infection (Fig. 3a–c and 4a–c) whereas such values were 0.514 and 0.497 for light (Fig. 3d and 4d) and heavy infection (Fig. 3e and 4e) at week 5 post-infection, respectively. The upwards and downwards peaks respectively denote the elevated and alleviated metabolites in the infected mice with hot colored (e.g. red) metabolites contributing more significantly to the class discrimination than cold colored ones. Blood plasma samples showed significant elevation of glucose and a range of amino acids together with depletion of lipoproteins and keto-bodies, including D-3-hydroxybutyrate and acetone after infection for 3 and 4 weeks (Fig. 3a–b). Compared to the controls, the metabolic changes of plasma obtained from the lightly infected mice (Fig. 3d) at week 5 post-infection were similar to those obtained from the mice at week 4 post-infection (Fig. 3c) whereas additional metabolite changes were observed for the heavily infected mice at week 5 post-infection (Fig. 3e). Such changes included elevated levels of *N*-acetyl-glycoproteins and reduced levels of glucose, citrate (i.e., Krebs cycle intermediate) and choline metabolites (Fig. 3e) such as PC and GPC.

Compared with controls, the infected mice showed obvious urinary metabolic changes at week 3 post-infection with significant elevation of 2-keto-3-methyl-valerate, 2-keto-isovalerate, 3-UP and some gut-microbiota related metabolites, including TMA, trimethylamine-*N*-oxide (TMAO) and PAG together with alleviation of adipate and Krebs cycle intermediates (such as succinate, 2-oxoglutarate and citrate). As the disease progressing, elevated levels of 4-cresol glucuronide, pyruvate, dimethylamine, malonate, glycine, indoxysulfate, and decreased levels of α-hydroxyisobutyrate, acetate, taurine and hippurate were observed in the urine of infected mice. The urinary metabolic profiles obtained from the lightly infected mice at week 5 post-infection were characterized by the increased pyruvate, glycine, 3-UP and microbiota related metabolites, such as PAG, 4-cresol glucuronide and TMA (Fig. 4d). For the heavily infected group, additional elevation of 2-keto-3-methyl-valerate, 2-keto-isovalerate and creatine together with alleviation of hippurate, 2-(4-hydroxyphenyl) propanoic acid and citrate were noted (Fig. 4e).

The cross-validated O-PLS-DA was further conducted for the metabolic profiles of liver tissue obtained at week 5 post-infection. The color-coded coefficient plot (Fig. 5) indicated that compared to controls, the infected mice showed higher levels of hepatic lactate, choline, PC, GPC, TMAO, taurine, creatine and a range of amino acids together with lower levels of glucose and glycogen (Table 2).

### Correlation of ^1^H NMR data with worm burden

**Figure 6:**
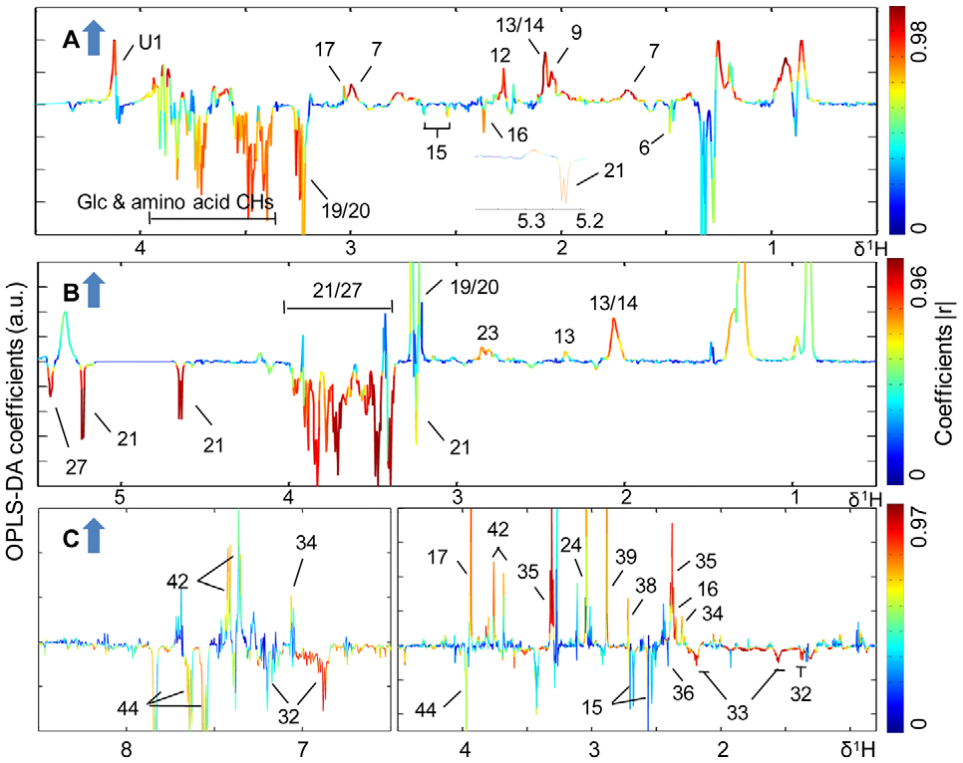
PLS correlation of the NMR spectral data of biofluids and liver tissues with the worm burden. PLS correlation plot derived from the CPMG filtered ^1^H NMR data for plasma (R^2^X = 0.521, Q^2^ = 0.281) (A), ^1^H HRMAS NMR data for liver tissue (R^2^X = 0.541, Q^2^ = 0.501) (B) and ^1^H NMR data for urine samples (R^2^X = 0.455, Q^2^ = 0.305) (C), which were all obtained from infected mice at week 5 post-infection, against worm burden. The arrows indicated increases in the severity of the infection.

Projection to latent structures (PLS) models were constructed using Pareto-scaled NMR data of plasma, liver and urine obtained at week 5 post-infection as corresponding X matrices and worm burden as Y matrix (Fig. 6). Significant correlations were found between the worm burden and metabolic changes in plasma (Fig. 6A), liver tissues (Fig. 6B) and urine samples (Fig. 6C). In plasma, reduced levels of glucose, pyruvate and increased levels of lysine and *N*-acetyl glycoprotein were closely associated with the worm burden (Fig. 6A) whilst, in the liver, reduced levels of glucose, glycogen and increased level of glutamate, glutamine were related to worm burden (Fig. 6B). In urine samples, the worm burden was associated with the elevated levels of PAG, 3-UP, creatine, 4-cresol glucuronide, TMAO and DMA together with alleviated levels of hippurate, 2-(4-hydroxyphenyl) propanoic acid and adipate (Fig. 6C).

**Figure 7:**
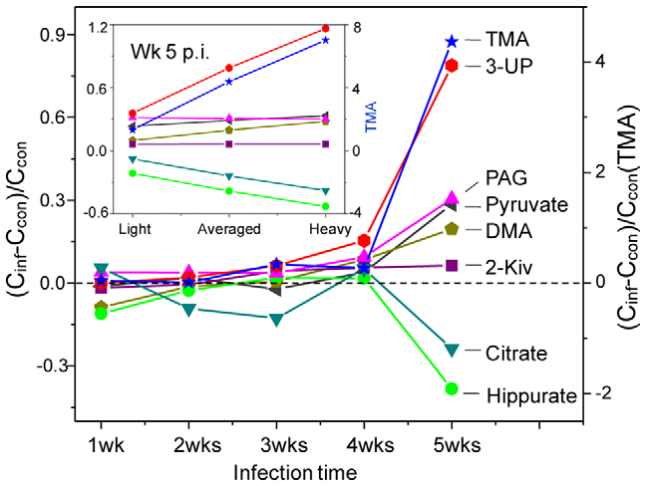
Metabolite concentration changes relative to corresponding controls at different time points after *S. japonicum* infection. The insert showed different severity at week 5 post infection. Keys: 3-UP, 3-ureidopropionate; PAG, phenylacetylglycine; 2-Kiv, 2-keto-isovalerate; DMA, dimethylamine; TMA, trimethylamine. C_inf_ and C_con_ stand for the averaged concentration in the infection and control group, respectively. Dashed lines indicated no changes and solid lines were for visual guidance only.

Fig. 7 shows the alterations of relative concentrations of typical metabolites as a function of infection duration and worm burden. It is apparent that infection causes steady increases in the concentrations of 3-UP, PAG and pyruvate together with decrease in the concentration of citrate. The elevation of 3-UP starts from week 3 post-infection and is positively correlated with infection duration and worm burden. Marked changes occurred at the fourth week post-infection for 3-UP, gut microbiota related metabolites (PAG, hippurate, DMA and TMA), pyruvate and citrate.

## Discussion

Above results indicated that metabolic changes in plasma, urine and liver tissues were all closely associated with the presence and severity of *S. japonicum* infection as indicated by the levels of worm burden. This work also comprehensively described the time-course metabolic responses of mice to schistosome infection with particular emphasis on the possibility of early detection of such infection.

### Metabolic response of infection at early stage

Previous investigations of metabolic response of schistosomal infections have been focused on the end point of one schistosome life cycle [28]–[30] when eggs have been produced. The metabolic responses of the same host to schistosome infection over time, starting from an early stage of infection have not been previously studied. Our investigation clearly showed that the schistosomal infection-induced metabolic changes were detectable from the third week post-infection in both plasma and urine samples (Fig. 3A and 4A). Such detection of infection is achieved one week earlier than the current “gold standard” method. *S. japonicum* worms reach maturity and begin to lay eggs around 4 weeks post-infection [44]–[45]. In the current study, the variations in metabolic profiles induced by the infection occurred before sexual maturation of *S. japonicum* worms in the mammalian host and thus prior to liver injuries by deposition of schistosomal eggs. Infection severity was also distinguished based on the metabolic profiles of both urine and plasma (Fig. 3C and 4C). Furthermore, metabolic profiles of plasma, urine and liver tissues are highly correlated with the intensities of worm burden (Fig. 6 and Fig. 7). These findings imply that metabonomic investigations of blood plasma and urine are potentially useful in the development of an early diagnostic tool for *S. japonicum* infection and assessment of infection severity.

### *S. japonicum* infection induced dynamic metabonomic changes

Liver injury is one of the most important manifestations of *S. japonicum* infection in humans. Our clinical chemistry data (Table 1) and histological results (Fig.S1) confirmed the occurrence of liver injuries at week 5 post-infection, being consistent with a previous human investigation [46]. One of the metabolic consequences of liver injury is the disturbance of amino acid metabolism, resulting in accumulation of amino acids in the liver and their depletion in plasma. In fact, such disturbed amino acid metabolism was previously noted with high levels of alanine, asparagine, creatine, glutamine and glycine in the *S. mansoni* infected mice liver [30]. These changes are also broadly similar to the increased concentrations of glutamine and glutamate relative to the lipids resulting from liver injuries caused by chronic hepatitis [47]. Our metabonomic results are clearly consistent with these observations, indicating that there might be some commonality for the metabolic responses to liver injuries caused by different schistosome species.

The accumulation of taurine observed here in the liver of schistosomal infected mice is probably due to the liver injury caused deficiency in the formation of taurine-conjugated bile acids and thus subsequent malabsorption that has been reported in human infected with schistosomes [1]. Since taurine is also a cell membrane stabilizer to maintain osmosis [48], its over-representation in liver may also reflect the liver cell membrane abnormalities following infection. Such view is further supported by the infection-induced accumulation of the cell membrane components (GPC and PC) in the host liver observed in current *S. japonicum* and previous *S. mansoni* infections [30].

Another important consequence of liver injury is stimulated glycolysis, which is manifested by marked reduction in levels of plasma glucose, liver glucose and glycogen, and the accumulation of liver lactate and urinary pyruvate following 5 weeks infection. Such stimulated glycolysis has also been observed for mice with *S. mansoni* infection for 49 days [28]. However, we further observed significant elevation of the plasma glucose for the infected mice at week 3 and 4 post-infection (Fig. 3a–b) and the light-infection group at week 5 post-infection (Fig. 3d). Such observation is broadly agreeable with the results of a previous study [49] on *S. mansoni* infection that hyperglycemia was observed for mice at the early stage. This is probably due to active manipulation and adaptation of parasites to the host rather than consequences of host injuries.

*S. japonicum* infection further led to the TCA cycle suppression with alleviation of plasma citrate and urinary citrate, 2-oxoglutarate and succinate which was similar to previous observations for *S. mansoni* infection [28]–[29]. Such changes were also consistent with the previous findings that the expression of TCA cycle associated enzymes decreased about 5 folds after *S. mansoni* infection for 8 weeks [8]. Furthermore, the urinary 2-keto-isocaproate, 2-keto-3-methyl-valerate and 2-keto-isovalerate were degradation products of leucine, isoleucine and valine respectively. The elevations of these keto acids observed here indicated that *S. japonicum* infection promoted ketogenesis resulting from the degradations of the branched-chain amino acids. Such effects appeared to be similar to the infection by another schistosome species *S. mansoni* [28].

The marked reduction of lipoproteins observed in the plasma of *S. japonicum* infected mice here is broadly consistent with the results from a proteomic study [9], which has shown that *S. japonicum* absorbs up to fifty host proteins. The reduction of lipoproteins can further be explained by the ability of adult schistosomes to take up the host phospholipids and triacylglycerols [50] to form a lipid tegument, which accounts for about one-third of the adult schistosomes and plays an important role in evading the host immune systems [51]. Therefore, the observed reduction of lipoproteins in the plasma of infected mice is probably a common consequence of worm developments for both *S. japonicum* and *S. mansoni* species [52] in both rodents and human [53] as well.

Moreover, the infection-induced changes of in gut microbiota related metabolites such as PAG, hippurate, TMA and DMA (Table 3) indicated that schistosome infection also disturbed the gut microbial ecology. This was broadly similar to the effects of infection by *S. mansoni* [28]–[29], suggesting such effects as a universal consequence of schistosomiasis. Amongst the microbial related metabolites, elevations of 4-cresol glucuronide and PAG and depressed levels of hippurate appeared to be common for the hosts infected with helminths [28]–[29] and intestinal nematodes [54]. Further research is required to determine the fine-grained alterations in the microbial community associated with infection, which will enhance our understanding of three-way host-parasite-microbiota interactions.

In this study, elevation of urinary 3-UP was found in mice infected with *S. japonicum*. Such metabolite has also been found recently in the urine samples of mice 53 days after infection by *S. mansoni* with the combination of NMR and capillary electrophoresis methods [55]. However, our detection of urinary 3-UP in mice at the third week of *S. japonicum* infection was four weeks earlier than that in the previous investigation. In addition, the levels of 3-UP was positively correlated with disease progression and worm burden (Fig. 7). Therefore, urinary 3-UP could be a potential biomarker for early diagnosis of schistosome infection. Elevated urinary 3-UP has previously been found in a case of inborn error of β-ureidopropionase deficiency [56] and reported to be a neuro-toxin [57]. The elevated urinary 3-UP in this study suggested that the schistosomal infection caused reduction of β-ureidopropionase activity thus disturbed uracil metabolism. Such alterations are also reflected with the elevation of another uracil metabolite, malonate, after infection for five weeks. The presence and specificity of 3-UP is warranted for further verification as an early diagnostic biomarker in the schistosome infected humans and other animals.

In conclusion, metabonomic analyses of urinary and plasma samples were effective, with little or no invasiveness, in detecting *S. japonicum* infection to mice one week prior to the “gold standard” method and in distinguishing the severity of such infections in terms of worm-burdens. A good correlation of elevation of the urinary 3-UP is clearly evident with worm burden and progression. The overall metabonomic changes in plasma, urine and liver of infected animals were also associated with the time-course of *S. japonicum* infection. Most of the metabolic responses to *S. japonicum* infection were broadly similar to what previously observed to *S. mansoni* infection indicating the generic metabolic consequences of schistosomiasis. We further discovered the alterations of pyrimidine and lipid metabolisms induced by schistosome infection. The changed metabolites in the plasma and liver coincided with the schistosome development and were consistent with the metabolic signature of the early stage of liver damage. Our findings on mechanisms of host-parasite interaction in the disease process over time provide a new basis for development of an early diagnosis tool. Further investigation of metabolic alterations due to parasitic infections in humans is necessary to evaluate the specificity of the altered metabolites in human populations.

## Supporting Information

Alternative Language Abstract S1 Translation of the abstract into Chinese by Junfang Wu.
(0.03 MB DOC)

Figure S1 Histopathological results of liver from a non-infected mouse (A) and a mouse infected with *S. japonicum* for 5 weeks (B) (200 times). The arrows on the right hand side slice showed *S. japonicum* eggs.
(3.35 MB TIF)

Figure S2 PCA trajectory plots of plasma (A) and urine (B) data obtained from the mean PC1 and PC2 values of the *S. japonicum* infected mice (red) and its corresponding controls (blue) at indicated time point with error bars representing the standard deviations.
(0.72 MB TIF)

Figure S3 Plots of permutation tests (n = 200) for plasma profiles of controls and infected mice at pre-infection (A), at week 4 post-infection (B), control and heavily-infected mice at week 5 post-infection (C), and for liver profiles from control and infected mice at week 5 post-infection (D). R^2^ describes how well the derived model fits the data; Q^2^ describes the predictive ability of the derived model. Intercept values: (A) R^2^: 0.215, Q^2^: −0.107; (B) R^2^: 0.255, Q^2^:−0.122; (C) R^2^: 0.228, Q^2^: −0.251; (D) R^2^: 0.305, Q^2^: −0.253.
(0.91 MB TIF)

Figure S4 Permutation tests (n = 200) for models obtained from urinary profiles of controls and infected mice at pre-infection (A), week 3 post-infection (B), week 4 post-infection (C) and from control and heavily-infected mice at week 5 post-infection (D). Intercept values are shown on each plot. Intercept values: (A) R^2^: 0.355, Q^2^: −0.144; (B) R^2^: 0.297, Q^2^: −0.249; (C) R^2^: 0.277, Q^2^: −0.198; (D) R^2^: 0.311, Q^2^: −0.253.
(0.17 MB TIF)

Text S1 Description of permutation test. Figures: Histopathological results of liver from a control mouse and a *S. japonicum* infected mouse for 5 weeks. PCA trajectory plot of plasma and urine spectra obtained from the *S. japonicum* infected mice and their corresponding control at different time point. The results obtained from the permutation tests for NMR data obtained from plasma at several time points and liver. The results obtained from the permutation tests for NMR data obtained from urine at several time points.
(0.03 MB DOC)
