# Supplementary material for: Metabolic Changes Reveal the Development of Schistosomiasis in Mice
Source: PLoS Negl Trop Dis. 2010 Aug 31;4(8):e807. doi: 10.1371/journal.pntd.0000807 (PMC2930859; doi:10.1371/journal.pntd.0000807)
Supplement: Alternative Language Abstract S1 — Translation of the abstract into Chinese by Junfang Wu. (0.03 MB DOC) [file pntd.0000807.s001.doc]

**小鼠在感染血吸虫病的发病过程的代谢变化**

**Junfang Wu^1,2^, Wenxin Xu^1,2^, Zhenping Ming^3^, Huifen Dong^3^, Huiru Tang^1*^, Yulan Wang^1*^**

**1** State Key Laboratory of Magnetic Resonance and Atomic and Molecular Physics, Wuhan Center for Magnetic Resonance, Wuhan Institute of Physics and Mathematics, Chinese Academy of Sciences, Wuhan, 430071, P.R. China, **2** Graduate School of Chinese Academy of Sciences, Beijing, P.R. China, **3** Department of Medical Parasitology, School of Basic Medical Science, Wuhan University, Wuhan, P.R. China.

血吸虫病是由裂体吸虫引起的一种人畜共患的感染性疾病，其中，日本血吸虫病在亚洲广为流行。为了认识血吸虫感染对宿主代谢组的影响，进而为疾病的早期诊断提供依据，我们运用以核磁共振为基础的代谢组学检测技术结合多变量统计数据分析方法，分析了日本血吸虫感染后每周（共五周）小鼠体液和感染后第五周肝脏的代谢组的动态变化特征。发现在感染后第三周即可从小鼠的血液及尿液中检测到血吸虫病的发生，表明代谢组学方法较金标准的检测方法提早一周。感染引起宿主代谢组的变化主要包括尿嘧啶分解代谢产物如3-脲基丙酸的显著增加、脂类代谢的异常、糖酵解的促进、三羧酸循环的抑制以及宿主肠道菌群微生态的紊乱。此外，研究还发现3-脲基丙酸等尿样代谢物及血浆、肝脏代谢组的变化与感染的发展和感染程度密切相关。以上结果使我们对感染后宿主的代谢应答有了更进一步的认识，并表明代谢组分析方法有可能为血吸虫病早期诊断提供有用的信息，也为深入理解疾病发展机制提供了重要的基础。
