# Supplementary figures and images for: Metabolic Changes Reveal the Development of Schistosomiasis in Mice
Source: PLoS Negl Trop Dis. 2010 Aug 31;4(8):e807. doi: 10.1371/journal.pntd.0000807 (PMC2930859; doi:10.1371/journal.pntd.0000807)

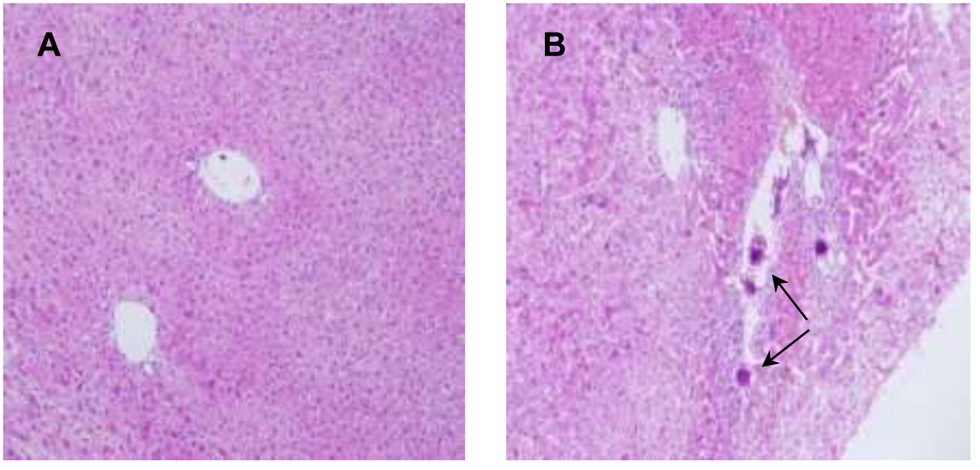

Supplement: Figure S1 — Histopathological results of liver from a non-infected mouse (A) and a mouse infected with S. japonicum for 5 weeks (B) (200 times). The arrows on the right hand side slice showed S. japonicum eggs. (3.35 MB TIF) [file pntd.0000807.s002.tif]

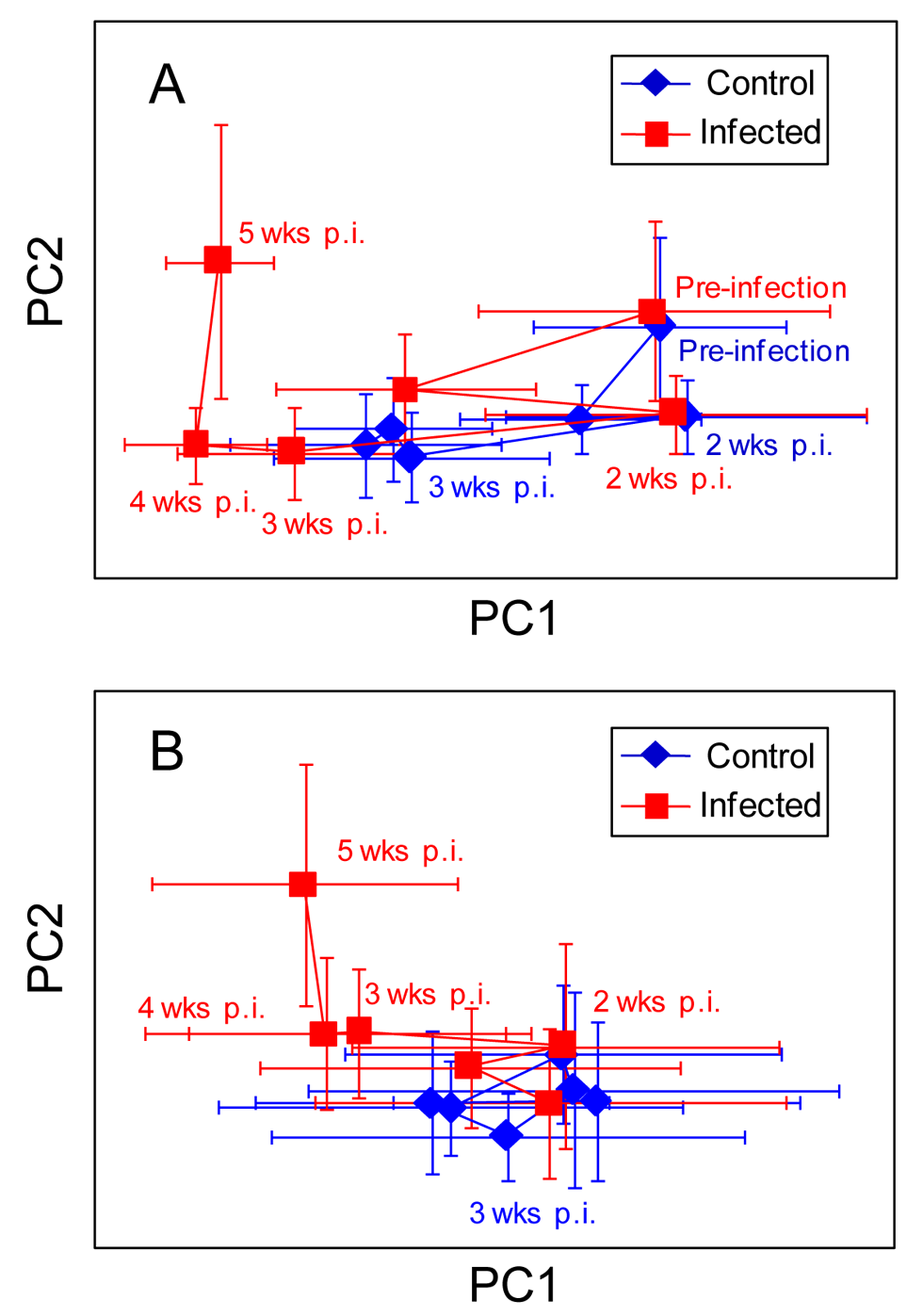

Supplement: Figure S2 — PCA trajectory plots of plasma (A) and urine (B) data obtained from the mean PC1 and PC2 values of the S. japonicum infected mice (red) and its corresponding controls (blue) at indicated time point with error bars representing the standard deviations. (0.72 MB TIF) [file pntd.0000807.s003.tif]

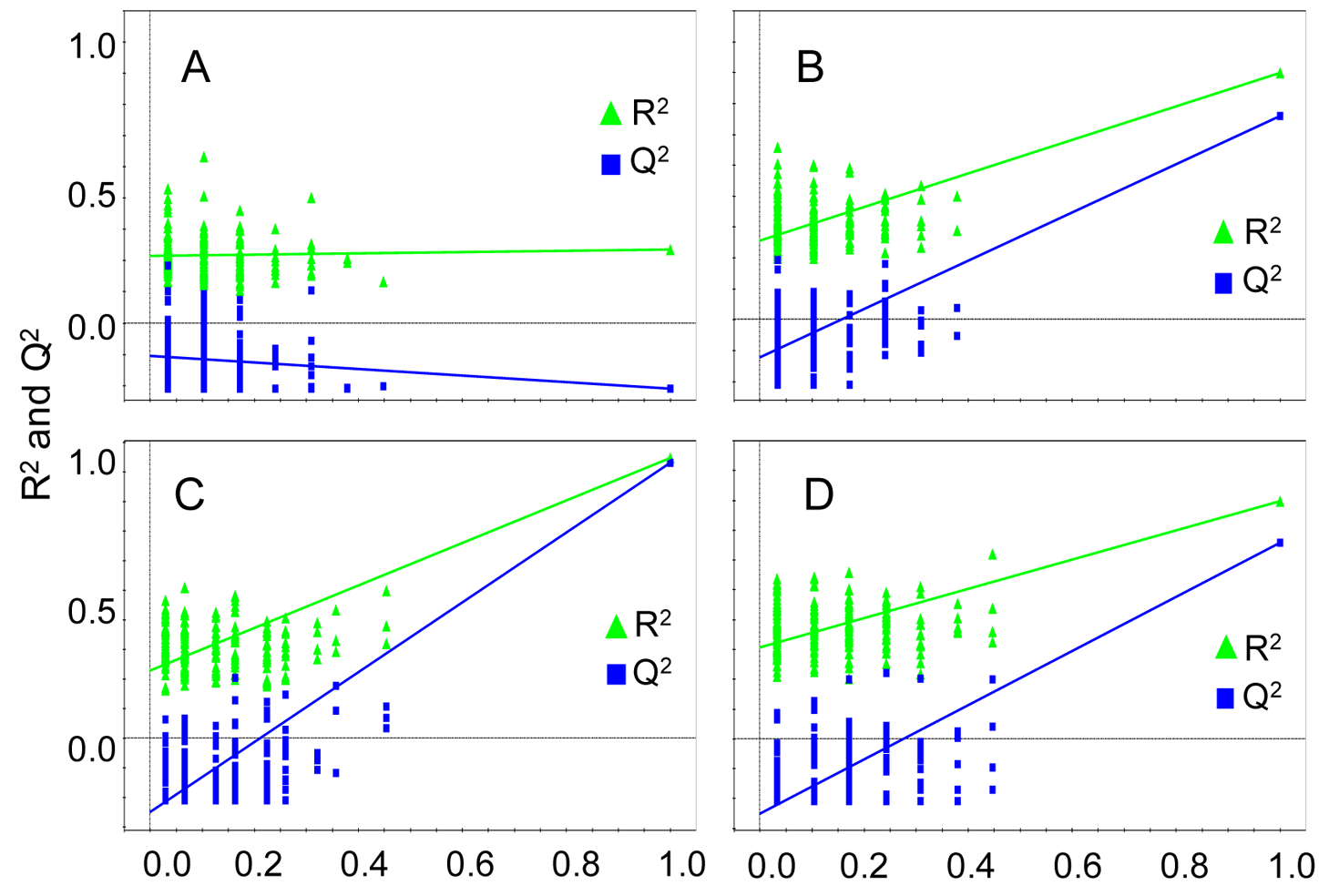

Supplement: Figure S3 — Plots of permutation tests (n = 200) for plasma profiles of controls and infected mice at pre-infection (A), at week 4 post-infection (B), control and heavily-infected mice at week 5 post-infection (C), and for liver profiles from control and infected mice at week 5 post-infection (D). R2 describes how well the derived model fits the data; Q2 describes the predictive ability of the derived model. Intercept values: (A) R2: 0.215, Q2: −0.107; (B) R2: 0.255, Q2:−0.122; (C) R2: 0.228, Q2: −0.251; (D) R2: 0.305, Q2: −0.253. (0.91 MB TIF) [file pntd.0000807.s004.tif]

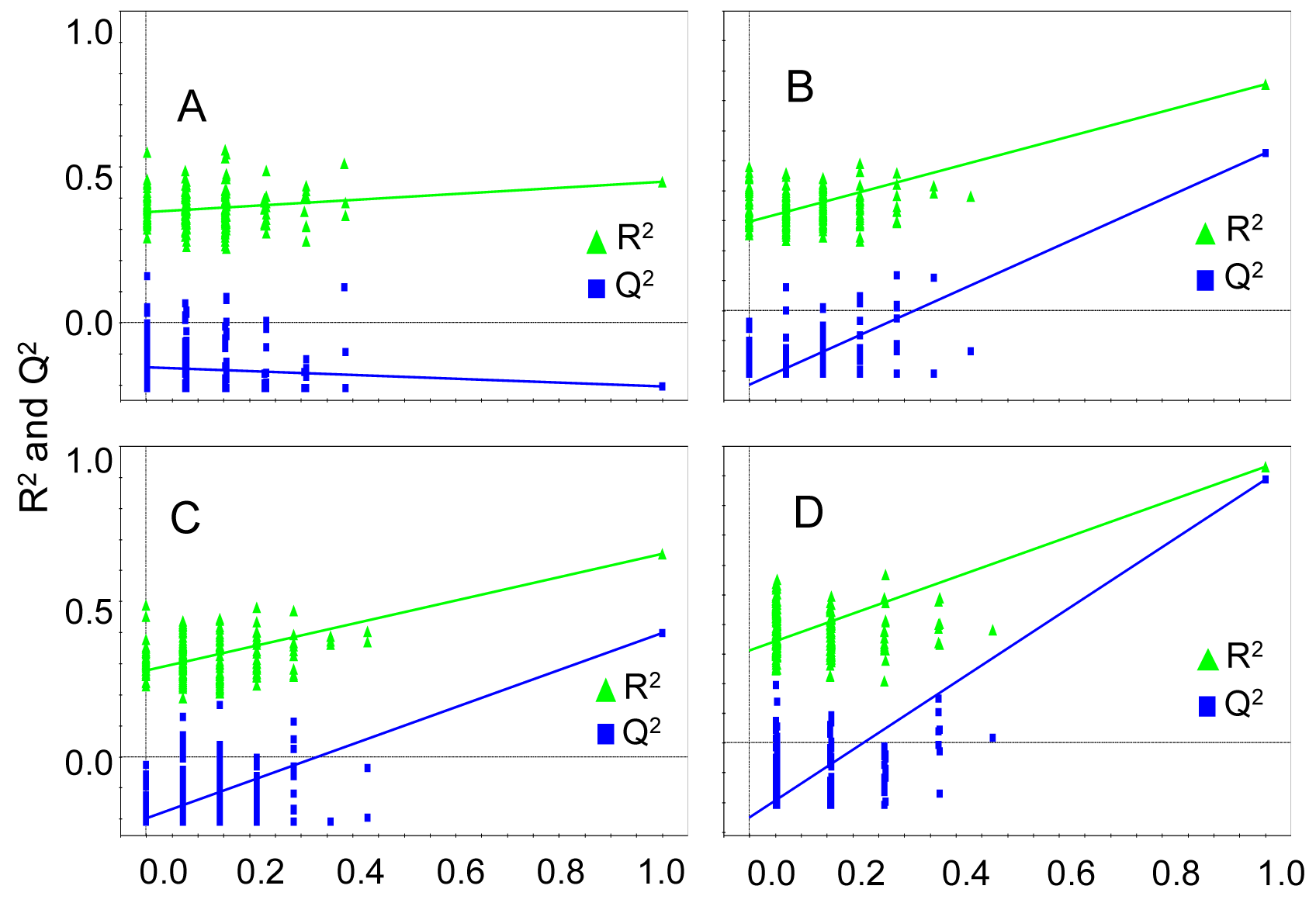

Supplement: Figure S4 — Permutation tests (n = 200) for models obtained from urinary profiles of controls and infected mice at pre-infection (A), week 3 post-infection (B), week 4 post-infection (C) and from control and heavily-infected mice at week 5 post-infection (D). Intercept values are shown on each plot. Intercept values: (A) R2: 0.355, Q2: −0.144; (B) R2: 0.297, Q2: −0.249; (C) R2: 0.277, Q2: −0.198; (D) R2: 0.311, Q2: −0.253. (0.17 MB TIF) [file pntd.0000807.s005.tif]
