# Supplementary material for: Metabolic Changes Reveal the Development of Schistosomiasis in Mice
Source: PLoS Negl Trop Dis. 2010 Aug 31;4(8):e807. doi: 10.1371/journal.pntd.0000807 (PMC2930859; doi:10.1371/journal.pntd.0000807)
Supplement: Text S1 — Description of permutation test. Figures: Histopathological results of liver from a control mouse and a S. japonicum infected mouse for 5 weeks. PCA trajectory plot of plasma and urine spectra obtained from the S. japonicum infected mice and their corresponding control at different time point. The results obtained from the permutation tests for NMR data obtained from plasma at several time points and liver. The results obtained from the permutation tests for NMR data obtained from urine at several time points. (0.03 MB DOC) [file pntd.0000807.s006.doc]

**List of Supplementary Online Material**

Description of permutation test

**Figures:**

Histopathological results of liver from a control mouse and a *S. japonicum* infected mouse for 5 weeks.

PCA trajectory plot of plasma and urine spectra obtained from the *S japonicum* infected mice and their corresponding control at different time point.

The results obtained from the permutation tests for NMR data obtained from plasma at several time points and liver.

The results obtained from the permutation tests for NMR data obtained from urine at several time points.

**Results**

**Validation of the O-PLS-DA models.** The O-PLS-DA models generated from NMR data of plasma and urine at a different time points and the NMR data of liver tissues at the end of experiment were further validated using permutation test where a total of 200 models were calculated by randomizing the order of Y variable. The Q^2^s, describing the predictive ability of the model, and R^2^s, indicating the goodness of the fitting, produced from the permutation test [1, 2] are plotted together with the Q^2^ and R^2^ obtained from real model in the supplementary Figure S3 and S4. The model obtained from the comparison between infected mice and control mice at pre-infection day was invalid since the Q^2^s and R^2^s generated from the permuted Y variables are higher than those from the real model (Fig. S3A). This conclusion is also manifested by a flat regression line and a small R^2^ and Q^2^ (R^2^ = 0.21, Q^2^ = -0.11).

**References**

1. Clayton TA*, et al.* (2006) Pharmaco-metabonomic phenotyping and personalized drug treatment*.* *Nature* 440: 1073-1077.

2. Slupsky CM*, et al.* (2007) Investigations of the effects of gender, diurnal variation, and age in human urinary metabolomic profiles*.* *Anal Chem* 79: 6995-7004.
